# Platelets of patients with chronic kidney disease demonstrate deficient platelet reactivity *in vitro*

**DOI:** 10.1186/1471-2369-13-127

**Published:** 2012-09-28

**Authors:** Esther R van Bladel, Rosa L de Jager, Daisy Walter, Loes Cornelissen, Carlo A Gaillard, Leonie A Boven, Mark Roest, Rob Fijnheer

**Affiliations:** 1 Department of Clinical Chemistry and Hematology, University Medical Center Utrecht, Heidelberglaan 100, 3584, CX, Utrecht, The Netherlands; 2 Department of Internal Medicine and Laboratory Medicine, Meander Medical Center, Utrechtseweg 160, 3800, BM, Amersfoort, The Netherlands; 3 Department of Nephrology, VU University Medical Center, Amsterdam, The Netherlands

**Keywords:** Platelet activation, Haemodialysis, Cardiorenal syndrome, End-stage renal disease

## Abstract

**Background:**

In patients with chronic kidney disease studies focusing on platelet function and properties often are non-conclusive whereas only few studies use functional platelet tests. In this study we evaluated a recently developed functional flow cytometry based assay for the analysis of platelet function in chronic kidney disease.

**Methods:**

Platelet reactivity was measured using flow cytometric analysis. Platelets in whole blood were triggered with different concentrations of agonists (TRAP, ADP, CRP). Platelet activation was quantified with staining for P-selectin, measuring the mean fluorescence intensity. Area under the curve and the concentration of half-maximal response were determined.

**Results:**

We studied 23 patients with chronic kidney disease (9 patients with cardiorenal failure and 14 patients with end stage renal disease) and 19 healthy controls. Expression of P-selectin on the platelet surface measured as mean fluorescence intensity was significantly less in chronic kidney disease patients compared to controls after maximal stimulation with TRAP (9.7 (7.9-10.8) vs. 11.4 (9.2-12.2), P = 0.032), ADP (1.6 (1.2-2.1) vs. 2.6 (1.9-3.5), P = 0.002) and CRP (9.2 (8.5-10.8) vs. 11.5 (9.5-12.9), P = 0.004). Also the area under the curve was significantly different. There was no significant difference in half-maximal response between both groups.

**Conclusion:**

In this study we found that patients with chronic kidney disease show reduced platelet reactivity in response of ADP, TRAP and CRP compared to controls. These results contribute to our understanding of the aberrant platelet function observed in patients with chronic kidney disease and emphasize the significance of using functional whole blood platelet activation assays.

## Background

In chronic kidney disease both bleeding and thrombotic complications are observed. It has been hypothesized that this disturbed balance between pro- and anti-haemostatic factors is involved in the high morbidity and mortality reported in chronic kidney disease [1-4]. Early stages of chronic kidney disease are typically associated with a prothrombotic tendency, whereas in its more advanced stage patients also suffer from a bleeding diathesis [5].

Bleeding tendency of patients is characterized by haemorrhagic symptoms and by prolongation of bleeding time [6]. The cause of bleeding in this group of patients has been elaborated in the past and the pathogenesis seems multifactorial. It is suggested that abnormal platelet function is a major contributor, since haemorrhage occurs despite a coagulation profile of normal or elevated levels of coagulation factors and normal platelet counts [4,6]. Platelet dysfunction may be the result of decreased dense granule content, decreased sensitivity to platelet agonists, abnormal expression of platelet glycoproteins, defective arachidonate metabolism and depressed prostaglandin metabolism as well as impaired platelet adhesiveness. Platelet dysfunction is thought to be caused by the action of uremic toxins, anemia, increased nitric oxide production, von Willebrand factor abnormalities and the use of medication like aspirin, non-steroidal anti-inflammatory drugs and β-lactam antibiotics [4,6-8]. Besides an increased bleeding risk, a variety of thrombotic complications are observed in patients with chronic renal failure, including coronary heart disease, cerebrovascular disease, peripheral vascular disease and heart failure. Already in mild to moderate chronic kidney disease an increased risk of cardiovascular events and higher mortality have been reported [1,2,9-12].

The balance between thrombosis and bleeds is disturbed in chronic kidney disease. Functional platelet tests are needed to obtain more insight into this balance. However, up to know only a minority of the studies used functional platelet tests and results are non-conclusive [6,13]. This is probably due to *in vitro* artifacts as a result of the procedure of platelet isolation, influence of plasma proteins and influence of hematocrit. Therefore we have set up this study with a recently developed, flow cytometer based, functional platelet assay in whole blood, in which these factors are not of influence. With this assay we assessed platelet reactivity in patients with end stage renal disease and cardiorenal syndrome. Both platelet sensitiveness as well as maximal activation of platelets in response to 3 different platelet stimuli was measured, providing an explanation for the disturbed haemostatic balance in patients with renal failure.

## Methods

### Subjects

Patients were selected from the out-patient clinic of the Meander Medical Center in Amersfoort for this prospective, observational study. Two groups of patients with chronic kidney disease were recruited. The first group consisted of patients with end stage renal disease receiving hemodialysis [14]. In patients on hemodialysis, blood samples were collected before and also immediately after hemodialysis (pre versus post dialysis). All samples were collected using citrate tubes and mixed gently. The second group consisted of patients with cardiorenal syndrome, defined as coexistence of chronic kidney disease and chronic heart failure [15]. Chronic kidney disease in this group was defined as eGFR < 70 ml/min without requirement of hemodialysis. Chronic heart failure was defined as NYHA class II or higher, based on symptoms, signs and objective abnormality on echocardiography [16]. Controls included subjects recruited from among healthy hospital staff, healthy subjects attending the hospital for control follow up and healthy partners of patients. Patients were excluded from the study based on the following criteria: clinical signs of infection, malignancy, primary haemostatic disorders unrelated to uremia, treatment with immunosuppressive drugs, use of antiplatelet agents (except aspirin) such as clopidogrel, dipyridamole or non-steroidal anti-inflammatory drugs and the inability to provide informed consent. The protocol was approved by the local medical ethical committee of Meander Medical Center Amersfoort and both patients and control volunteers gave written informed consent to participate in the study.

### Blood sampling and processing

In patients on haemodialysis, the samples were collected before starting the haemodialysis and immediately after the procedure. The first sample was taken from the afferent line directly after inserting the needle in the fistula. The second sample was taken directly after dialysis from the efferent line out of the dialysis machine. In control subjects peripheral venous blood samples were collected from the antecubital vein using 21 gauge needles. A total of 4.5 mL blood was drawn into vacutainer tubes containing 0.5 mL 3.2% sodium-citrate solution as anticoagulant and mixed gently. Blood cell count assays were performed using a haematology analyzer (Sysmex, Etten-Leur, The Netherlands). Hemoglobin, hematocrit, and platelet count were noted.

## Materials

For our samples we used HEPES-buffered saline (HBS) containing 10 mM HEPES (BDH biochemical, UK), 150 mM NaCl (Sigma-Aldrich, Zwijndrecht, the Netherlands), 1 mM MgSO_4_ (Riedel de Haën, Hannover, Germany) and 5 mM KCl (Riedel de Haën), with a pH of 7.4. Fixation was done with a fixation buffer containing 0.2% formaldehyde (Calbiochem, Merck, Darmstadt, Germany) in 0.9% NaCL (Sigma-Aldrich). Used fluorochrome-labeled ligands were anti-CD42b fluorescein isothiocyanate (FITC)-labeled mouse antihuman antibody (BD Pharmingen™, San Diego, California, USA) and anti-CD62P phycoerythrin (PE)-labeled mouse antihuman antibody (BD Pharmingen™). Adenosine diphosphate (ADP) (Roche, Almere, the Netherlands) was used as agonist, as well as thrombin-receptor-associated-peptide (TRAP) (Bachem, Weil am Rhein, Germany) and cross-linked collagen related peptide (CRP) was a generous gift of R. Farndale (Cambridge, United Kingdom).

### Platelet reactivity

Assays were performed as described before [17]. In short, 5 μL of whole blood was added to tubes containing 50 μL of HBS, fluorochrome-labeled ligands and serial dilutions of agonists. Anti-CD42b FITC-labeled monoclonal antibodies were used as an activation-independent platelet marker. PE-labeled anti-CD62P was used as an activation-dependent marker. For each agonist eight different concentrations were used, with four times dilution steps between each sample. Concentrations of TRAP were 0.038 to 625 μmol/L, ADP 0.008 to 125 μmol/L, and CRP 0.2 to 2500 ng/mL. After incubation at room temperature for 20 minutes, platelets were fixed by the addition of 500 μL fixative (0.2% paraformaldehyde). Subsequently, samples were 3.5 times diluted with the same fluid for flow cytometric analysis. Samples were analyzed by flow cytometry on a Epics XL-MCL Flow Cytometer (Beckman Coulter, Miami, Florida, USA) and EXPO 32 MultiCOMP Software (Beckman Coulter) was used to process the data. The platelet population was identified by forward and 90° side scatter properties in combination with a positive CD42b signal. Isotype control antibodies were used to correct for aspecific binding. The mean fluorescence intensity (MFI) of all platelets is expressed in arbitrary units (AU).

### Statistical analysis

GraphPad software version 4.0 for Windows (GraphPad Software, San Diego, California USA) was used to draw graphs and to calculate EC50. EC50 is defined as the concentration of the agonist needed to achieve an effect on the platelets halfway between the maximum and minimum. Area under the curve (AUC) was calculated in SPSS by adding up the outcome minus basal activation outcome of all concentrations points. The data were analyzed by logistic regression for scaled data. For comparisons of nominal variables a Chi-square cross tabulation with a Fisher’s Exact test was used. Statistical analyses were undertaken using SPSS software version 15.0 (SPSS Inc, Chigaco, Illinois). A P-value of < 0.05 was considered as statistically significant. Values are given as median with interquartile range (IQR) if not noted otherwise.

## Results

### Patient characteristics

Twenty-three patients with chronic kidney disease and 19 healthy controls were included. In the chronic kidney disease group, we included 9 patients with cardio renal syndrome and 14 patients with end stage renal failure on hemodialysis (analyzed before and immediate after dialysis). Clinical and laboratory characteristics are listed in Table 1. Age was significantly higher in patients (78 years (64-83)) compared to controls (62 years (48-71)). As expected hemoglobin and hematocrit level were significantly lower in cardio renal syndrome patients compared to controls. There were no significant differences in platelet count. The etiology of chronic kidney disease was divers: hypertension (n = 7), diabetic nephropathy (n = 4), membranous glomerulopathy (n = 1), rapid progressive IgA nephropathy (n = 1), chronic pyelonephritis (n = 1), polycystic nephropathy (n = 1), nephrosclerosis (n = 1) or unknown cause (n = 7). The etiology of chronic heart failure was of ischemic origin (n = 5), valvular heart disease (n = 3) or unknown (n = 1).

**Table 1:** **Baseline characteristics in patientswith chronic kidney diseaseand controls**

**Baseline characteristics**	**Controls**	**CKD**	**P-value**
	**N = 19**	**N = 23**	
Age, y (IQR)	62 (48-71)	78 (64-83)	0.043
Male, n (%)	13 (68.4)	15 (65.2)	0.755
Hemoglobin, mmol/L (IQR)	9.4 (9.0-9.9)	7.2 (6.7-7.8)	0.006
Hematocrit, L/L (IQR)	0.44 (0.43-0.47)	0.36 (0.34-0.38)	0.001
Platelet count, 10^9^/L (IQR)	210 (150-240)	187 (160-225)	0.223
Hemodialysis, n (%)	NA	14 (60.9)	-
Cardiorenal syndrome, n (%)	NA	9 (39.1)	-
EPO use, n (%)	NA	5 (21.7)	-
Aspirin use, n (%)	0	11 (47.8)	-

*CKD* chronic kidney disease, *IQR* inter quartile range, *EPO* erythropoietin.

### Platelet reactivity

**Figure 1:**
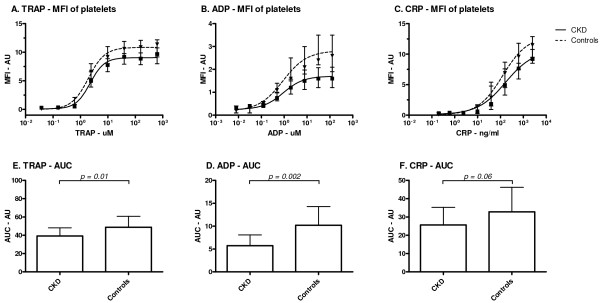
**Platelet reactivity. A**: The mean fluorescence intensity (MFI) of all platelets, expressed in arbitrary units (AU) and **B**: area under the curve (AUC), displayed as median plus interquartile range, for chronic kidney disease patients (CKD) and controls.

Expression of P-selectin on the platelet surface measured as MFI was significantly lower in chronic kidney disease patients compared to controls after maximal stimulation with TRAP (9.7 (7.9-10.8) vs. 11.4 (9.2-12.2), P = 0.032), ADP (1.6 (1.2-2.1) vs. 2.6 (1.9-3.5), P = 0.002) and CRP (9.2 (8.5-10.8) vs. 11.5 (9.5-12.9), P = 0.004). (Figure 1 and Table 2). Maximal P-selectin expression in response to different agonist correlated significantly with each other (Spearmann’s Rho correlation TRAP and ADP 0.684 with *p* < 0.001; TRAP and CRP 0.538 with *p* < 0.001; ADP and CRP 0.475 with *p* = 0.001). Table 2 shows that the EC50 was not different between chronic kidney disease patients and controls.

**Table 2:** Results platelet activation

	**Controls**	**CKD**	**P-value**	**P-value**
	**N = 19**	**N = 23**		**Adjusted for age**
Maximal MFI TRAP, AU (IQR)	11.4 (9.2-12.2)	9.7 (7.9-10.8)	0.032	0.095
Maximal MFI ADP, AU (IQR)	2.6 (1.9-3.5)	1.6 (1.2-2.1)	0.002	0.004
Maximal MFICRP, AU (IQR)	11.5 (9.5-12.9)	9.2 (8.5-10.8)	0.004	0.010
EC50 TRAP, μM (IQR)	1.9 (1.5-2.5)	2.3 (1.8-2.6)	0.372	0.182
EC50 ADP, μM (IQR)	0.9 (0.6-1.1)	0.89 (0.7-1.1)	0.544	0.597
EC50 CRP, ng/ml (IQR)	120 (46.7-312.7)	116.1 (46.1-270.5)	0.955	0.660
AUC TRAP, AU (IQR)	47.1 (41.1-54.2)	39.5 (32.9-44.4)	0.012	0.023
AUC ADP, AU (IQR)	8.9 (7.9-12.2)	5.8 (3.8-7.3)	0.002	0.003
AUC CRP, AU (IQR)	30.3 (22.1-41.9)	23.4 (18.9-34.7)	0.063	0.322

*MFI* Mean fluorescence intensity, *AU* arbitrary units, *IQR* inter quartile range, *EC50* concentration of agonist leading to a half maximal P-selectin expression, *AUC* Area under the curve.

Chronic kidney disease patients showed significantly lower platelet reactivity as compared to healthy control subjects for TRAP (AUC : 39.5 (32.9-44.4) vs. 47.1 (41.1-54.2), P = 0.01) and ADP (AUC: 5.8 (3.8-7.3) vs. 8.9 (7.9-12.2), P = 0.002) when measured with AUC (Table 2). Stimulation with CRP (AUC: 23.4 (18.9-34.79) vs. 30.3 (22.1-41.9), p = 0.06) does not show a difference in reactivity between the groups.

Since age was significant lower in the control group we adjusted for this in a regression analysis. The significant difference in MFI and AUC between chronic kidney disease patients and controls after maximal stimulation remained significant (Table 2). There was no significant difference between patients with cardiorenal failure and patients with end stage renal failure.

In the chronic kidney disease group almost half of the patients used aspirin as anti-platelet therapy. Platelet reactivity in this group does not show a difference between aspirin users and patients without aspirin. This accounts for all outcome measurements.

### Pre-versus-post dialysis

To rule out an activating effect of the hemodialysis procedure on baseline platelet activation we studied platelet activation before and immediate after dialysis. The platelet sensitivity (EC50) for TRAP, ADP and CRP did not differ between patients pre- and post dialysis. There was no difference between the maximum reached effect and AUC on platelet activation when comparing patients pre- and post dialysis (data not shown).

## Discussion

The aim of this study was to investigate the relation between platelet function and kidney failure in patients with end stage renal disease and cardiorenal syndrome using a flow cytometer based, functional platelet assay. We show that P-selectin expression after stimulation with ADP, CRP and TRAP is lower in patients with chronic kidney disease as compared to healthy controls. Furthermore, we demonstrate that the EC50 was not different between groups. This means that platelet sensitivity itself is not affected for the different agonists, but the maximal platelet response is significantly lower.

Platelets were activated with 3 different stimuli. We chose for the agonists TRAP, ADP and CRP, which stimulate the three major physiological platelet activation pathways. TRAP activates the thrombin receptor Proteinase Activated Receptor 1 (PAR-1) on platelets. ADP is normally present in platelet dense granules. Upon platelet activation, ADP is released to activate nearby platelets via the P2Y receptors. CRP activates the receptor glycoprotein VI, the major collagen receptor on platelets. So, all 3 stimuli are of physiological importance.

P-selectin is found in the α-granula of platelets. A deficiency in α-granula could lead to ineffective haemostasis. Two different explanations are possible for the deficient platelet α-granula release found in chronic kidney disease. This could be due to depletion of α-granula itself, or due to a deficiency in the release of α-granula. An impaired α-granule release has already been reported in uraemia [18]. This is further supported by the recent observations of Schoorl et al. of an increase in platelets depleted from granules in patients undergoing chronic haemodialysis [19]. Nevertheless, we cannot exclude an impairment of release.

The clinical bleeding tendency in uremic patients received much attention [7]. Haemo- or peritoneal dialysis was found to improve haemostasis without correcting platelet aggregation defects. In contrast, compensatory mechanisms in the form of high von Willebrand factor (VWF) levels preserved relatively normal adhesion of uremic platelets to injured vessel wall models [20]. Moreover, as soon as the hematocrit - determining the red blood cell-mediated radial transport of blood platelets towards the vessel wall - was corrected by recombinant erythropoietin, clinical bleeding became less prominent. In contrast, thromboembolic/cardiovascular morbidity remains an important problem in these patients [21]. Especially in cardiorenal failure, there seems no or little benefit from treatment with antiplatelet agents, whilst risk of bleeding may increase [22]. Even though reports on platelet reactivity in patient with chronic kidney disease show conflicting results [23], it has been attributed to an acquired thrombocytopathy characterized by decreased aggregation of platelets in response to stimuli. There are only a few studies that used functional tests to study platelet function in chronic kidney disease. Aggarwal et. al. found a higher P-selectin expression in patients with end stage renal disease receiving haemodialysis compared to healthy controls after stimulation with a single concentration of ADP (0.2 μM). This suggests an increased reactivity [13]. Moal et. al performed a similar study in which ADP (200 μM) and TRAP (50 μM) in a single concentration were used to stimulate platelets in healthy controls and end stage renal disease patients receiving haemodialysis. They found a lower P-selectin expression in patients compared to controls, indicating reduced platelet reactivity in patients with chronic kidney disease [6]. Most assays used previously are influenced by *in vitro* artifacts, platelet isolation and are dependent on VWF or hematocrit. In our functional assay there is no role for VWF and hematocrit. Moreover, since fresh whole blood was used, and samples were fixated subsequent to stimulation, *in vitro* platelet activation was negligible. We couldn’t find an immediate effect of haemodialysis, but long term dialysis and classical risk factors for athero-vascular disease could all be studied with this assay. Larger populations should be studied in order to find the factors influencing platelet reactivity *in vivo*.

In our study, aspirin did not suppress the expression of P-selectin on platelets. Considering the fact that aspirin function is related to inhibition of tromboxane formation and does not influences release of granules, this is an expected result. Moreover, our finding is in accordance with a study by Stumpf et. al., in which P-selectin expression on platelets did not show a difference between patients taking aspirin compared to non-aspirin users [24]. Further investigation is required to determine the influence of different drugs on platelet reactivity.

We here demonstrate that the flow cytometry based platelet activation assay can be used in clinical practice to study variables influencing platelet response in patients with renal failure. The assay is based on incubation with different agonists in whole blood and subsequent fixation. Platelets are sensitive for *in vitro* artifacts (especially platelet isolation, shear stress, pH and temperature). In our assay platelets are not isolated. The incubation in whole blood under standardized condition and the subsequent fixation step makes *in vitro* artifacts unlikely. When repeating the assay within healthy subjects at different blood collection moments, we find high reproducibility. Moreover, this assay can be used in routine clinical practice.

## Conclusion

In conclusion, we found that patients with chronic kidney disease show reduced platelet reactivity in response of ADP, TRAP and CRP compared to controls. The defect is probably due to an α-granule defect. Further investigation is required to determine a correlation between platelet reactivity and both renal function and the high mortality risk of patients with chronic kidney disease.

## Competing interests

The authors declare that they have no competing interests.

## Author’s contributions

ERB participated in design of the study and interpretation of data, and contributed to drafting of the manuscript. RLJ participated in design of the study and in the performance of flow cytometry analysis, performed the statistical analysis and critically revised the manuscript. DW and LC carried out the FACS analysis, and participated in the statistical analysis of data and drafting of the manuscript. CAG, LAB and MR participated in the design of the study and critically revised the manuscript. RF designed the study, supervised its conduct and drafted the manuscript. All authors read and approved the final manuscript.

## Pre-publication history

The pre-publication history for this paper can be accessed here:

http://www.biomedcentral.com/1471-2369/13/127/prepub
